# Comparative Analysis of Chitin SynthaseA dsRNA Mediated RNA Interference for Management of Crop Pests of Different Families of Lepidoptera

**DOI:** 10.3389/fpls.2020.00427

**Published:** 2020-04-17

**Authors:** Seema Rana, Ashish B. Rajurkar, K. K. Kumar, Subbarayalu Mohankumar

**Affiliations:** Department of Plant Biotechnology, Centre for Plant Molecular Biology and Biotechnology, Tamil Nadu Agricultural University, Coimbatore, India

**Keywords:** *Spodoptera litura*, *Chilo partellus*, *Plutella xylostella*, *Maruca vitrata*, RNAi, *CHSA*

## Abstract

RNA interference (RNAi) is a sequence-specific down-regulation in the expression of a particular gene, induced by double-stranded RNA (dsRNA). Feeding of dsRNA either directly or through transgenic plants expressing dsRNA of insect genes has been proven successful against lepidopteran and coleopteran pests, establishing an additional alternative to control insect pests. Lepidopteran crop pests including *Spodoptera litura* (Fabricius) (Noctuidae), *Chilo partellus* (Swinhoe) (Crambidae), *Plutella xylostella* (Linnaeus) (Plutellidae), and *Maruca vitrata* (Fabricius) (Pyralidae) are the devastating pests of a variety of crops. To tap the potential of RNAi against insect pests, a gene coding for the key enzyme in chitin biosynthesis in arthropods, the *chitin synthaseA* (*CHSA*), has been targeted through an exogenous delivery of dsRNA and plant-mediated RNAi. The introduction of *dsCHSA* caused “Half ecdysis” and “Black body” type lethal phenotypes and a significant reduction in larval body weight. Subsequent RT-qPCR analysis demonstrated the down-regulation of *CHSA* gene transcripts from 1.38- to 8.33-fold in the four target species. Meanwhile, when *S. litura* larvae fed with leaves of transgenic tobacco plants expressing *dsSlCHSA*, the mRNA abundance of *CHSA* gene was significantly decreased resulting in lethal phenotypes like “Double head formation,” “Half ecdysis,” and “Black body.” In addition, abnormalities in pupal–adult and adult stage were also documented, strongly suggesting the RNAi effect of *CHSA* gene at late developmental stages. Overall, the results demonstrated that *CHSA* gene expression in Lepidopteran crop pests could be suppressed by application of dsRNA either as feeding or through transgenic crop plants.

## Introduction

Lepidopteran crop pests including tobacco cutworm, *Spodoptera litura* (Fabricius) (Lepidoptera: Noctuidae); maize/sorghum stem borer, *Chilo partellus* (Swinhoe) (Lepidoptera: Crambidae); diamondback moth, *Plutella xylostella* (Linnaeus) (Lepidoptera: Plutellidae), and legume pod borer, *Maruca vitrata* (Fabricius) (Lepidoptera: Pyralidae) are considered as most destructive pests of several economically important agricultural and horticultural crops worldwide. Significant amount of yield losses caused by those insect pests has been reported in several regions of world (Ahmad et al., 2008; Sharma et al., 2010; Zalucki et al., 2012). The most common method for the management of those insect pest is the use of insecticides or bio-control agents particularly *Bacillus thuringiensis* (Bt) toxins. However, the management of those insect pests is a daunting task because of the development of resistance against insecticides and Bt toxins (Storer et al., 2010). These problems necessitate finding an alternative pest control strategy to supplement the present pest management methods.

RNA interference (RNAi)-based strategy through the expression of double-stranded RNA (dsRNA) targeting potential genes in crop pests has paved the way for new generation of integrated pest management (IPM). RNAi is a conserved phenomenon in which a dsRNA knocks down the expression of a target gene. However, its efficiency varies with the target insect species (Terenius et al., 2011), the method of RNAi administration (Scott et al., 2013), and the candidate gene targeted by RNAi (Kola et al., 2015). In the preceding years, several successful RNAi experiments in lepidopterans have been reported and published. However, the comparative analysis to study the effect of RNAi among crop pests belonging to different families in lepidopterans has not been appeared so far.

The basic methods of dsRNA administration in insects are injection and feeding. Most of the RNAi experiments in different insect orders including lepidopterans were conducted through droplet feeding or microinjection, particularly in *Drosophila melanogaster* (Meigen) (Miller et al., 2008), *Tribolium castaneum* (Herbst) (Tomoyasu and Denell, 2004; Bai et al., 2011), *Acyrthosiphon pisum* (Harris) (Ye et al., 2019), and *Bombyx mori* (Linnaeus) (Hossain et al., 2008), *Spodoptera exigua* (Hubner) (Kim et al., 2015). Initially, the RNAi aimed as a functional genomic tool to elucidate the functions of genes. For example, Bettencourt et al. (2002) and Quan et al. (2002) observed phenotypic variations in embryos in *Hyalophora cecropia* (Linnaeus) and *B. mori*, after injection of dsRNA into the pupa demonstrating systemic RNAi. Chen et al. (2008) also observed abnormal larval growth and development in *S. exigua* after injection of dsRNA of *chitin synthaseA* (*CHSA*). Though the injection method showed phenotypic variations, the delivery of dsRNA through injection has low survival rate and is not feasible in the field conditions to control crop pests. Furthermore, Wang et al. (2011) demonstrated that a direct spray of dsRNA in *Ostrinia furnalalis* (Linnaeus) larvae, resulting in down regulation in the expression of target genes, delayed growth and development, and mortality. However, high amount of dsRNA is required to reach the threshold and produce the desired results, raising the question of specificity and cost effectiveness. Therefore, through the expression of dsRNA in plants involving transgenic plant-mediated RNAi is quite economical, its administration is easy and practicable in field conditions. Moreover, the transgenic plants expressing dsRNA of insect genes, to protect the plants against insect feeding damage have been proven successful against coleopteran and lepidopteran insect pests (Baum et al., 2007; Mao et al., 2007; Zha et al., 2011). However, to our knowledge, no studies have been documented deploying two different methods, both the direct application of dsRNA and plant-mediated RNAi by feeding to control crop pests of different families of lepidoptera.

Another important factor for the success of RNAi is the selection of potential target genes. The simplest and effective way is to select known ideal genes based on the literature. In the present study, we have selected *CHSA* as a target gene for dsRNA-transgenic plant-mediated RNAi. Chitin synthesis is essential for insect growth and development. Chitin, a polysaccharide of *N*-acetyl-B-D-glucosamine is an important component of insect cuticle which forms an exoskeleton (exo and endocuticle) and plays important role in protecting insects from environmental stresses and pathogenic microbes (Kramer and Muthukrishnan, 2004). The insect *chitin synthases* (*CHS*) are encoded by two genes, *CHSA*/*CHS1* and *CHSB*/*CHS2* (Hogenkamp et al., 2005). *CHSA* function exclusively in the formation of chitin found in the insect cuticle (epidermal and ectodermal cells), while *CHSB* function mainly in the formation of chitin in the peritrophic membrane of epithelial cells (Arakane et al., 2005; Wang et al., 2012). Moreover, chitin is mainly present in arthropods and absent in vertebrates and plants, could address an important concern of RNAi, i.e., the specificity of dsRNA (Tian et al., 2009). Significant promising results documenting various phenotypic abnormalities and lethality in *S. exigua* through disruption of *SeCHSA* by injection and bacterial expressed dsRNA of *SeCHSA* have been shown (Chen et al., 2008; Tian et al., 2009). However, the present comparative study using dsRNA-transgenic plant-mediated RNAi of *CHSA* in lepidopteran crop pests has proven new insights for designing futuristic pest management strategies.

In this study, *CHSA* gene was isolated, cloned, and sequences were compared from *S. litura*, *C. partellus*, *P. xylostella*, and *M. vitrata.* dsRNA of *CHSA* gene was synthesized and insect feeding study demonstrated its effect against the target species. We also developed hairpin RNAi construct targeting *CHSA* gene and transformed into tobacco plants. Both the direct feeding of dsRNA of *CHSA* gene and plant mediated RNAi demonstrated the significant reduction in larval body weight, higher lethality rate, and down-regulation in mRNA abundance of *CHSA* gene in all four lepidopteran insects studied.

## Materials and Methods

### Insects Studied

The larvae of *S*. *litura*, *P. xylostella*, and *M. vitrata* were collected from the research fields of Tamil Nadu Agricultural University, Coimbatore, and the eggs of *C. partellus* were obtained from the National Bureau of Agricultural Insect Resources (NBAIR), Bengaluru, Karnataka, India. They were reared on castor leaves (*Ricinus communis*) (Euphorbiaceae), cauliflower leaves (*Brassica oleracea* L.) (Brassicaceae), lablab pods (*Lablab purpureus* L.) (Fabaceae), and baby corn (*Zea mays*) (Poaceae), respectively, at the Molecular Ecology Laboratory, Department of Plant Biotechnology, Tamil Nadu Agricultural University, Coimbatore, India.

### Mass Culturing of Insects

Mass culturing was done with slight modification to the methodologies of Britto (1980) and described briefly here. The larvae hatched out from the eggs were reared on respective feed till pupation in plastic buckets (22.5 cm dia. and 25 cm height). The feed was changed once in 2 days during earlier stages and daily in later stages. The pupae were collected, surface sterilized with 0.5% sodium hypochlorite, rinsed with distilled water, and kept in an adult emergence cage. The newly emerged adults were transferred to plastic buckets for mating and oviposition and were fed with 10% sugar solution enriched with vitamin. Folded wax paper was placed inside the plastic buckets to lay the eggs. The temperature and relative humidity were maintained at 28 ± 3°C and 70–75%, respectively, inside the culture room.

### Cloning of *CHSA* Gene in *S. litura*, *C. partellus*, *P. xylostella*, and *M. vitrata*

#### RNA Isolation and cDNA Synthesis

Total RNA was extracted by homogenizing single third instar larvae of *S*. *litura*, *C. partellus*, *P. xylostella*, and *M. vitrata* individually by employing Trizol method (Chomczynski and Mackey, 1995). The isolated RNA was reverse transcribed using cDNA Synthesis Kit (Thermo Scientific, United States) after treating with RNase-free DNase I (Thermo Scientific, United States). The partial *CHSA* gene from *S. litura*, *C. partellus*, *P. xylostella*, and *M. vitrata* was amplified using a set of *CHSA* specific primers (Table 1). The PCR product was column purified as per the manufacture’s instruction provided by purification spin kit (BIOBASIC). The purified DNA fragments were used for cloning (pTZ57R/T vector, Thermo Scientific, United States) and bacterial transformation. Further validation of recombinant colonies was done by restriction digestion analysis and sequenced at SciGenom Labs Pvt. Ltd., Cochin, Kerala, India.

**TABLE 1:** Primers used in this study.

**Sl. No.**	**Primer name**	**Sequence (5′–3′)**	**Amplicon size (bp)**
1	CHSA	F-GTGATGATGATTCGCAAGTGA	518
		R-AGGATGAATACGACCGCAAG	
2	dsCHSA	F-taatacgactcactatagggGTGATGATGATTCGCAAGTGA	616
		R-taatacgactcactatagggAGGATGAATACGACCGCAAG	
3	S SlCHSA	F-CTCGAGGTGATGATGATTCGCAAGTGA	530
		R-GGTACCAGGATGAATACGACCGCAAG	
4	A SlCHSA	F-TCTAGAGTGATGATGATTCGCAAGTGA	530
		R-AAGCTTAGGATGAATACGACCGCAAG	
5	qRT CHSA	F-GACTCTGGACGGAGACAT	112
		R-GCCTACAGGATGAATACGAC	
6	qRT Actin	F-AATCGTGCGTGACATCAA	218
		R-TGTAAGTGGTCTCGTGGAT	

### Sequencing of Cloned Fragment and Analysis

The samples were sequenced through single pass analysis from forward and reverse direction. DNA sequence data was compared with available *CHSA* gene sequences in National Center for Biotechnology Information (NCBI) data bank^1^ by using BLASTn analysis tool. The sequences in different species were edited and aligned with reference sequences of *CHSA* (Accession No: JN003621.1) by ClustalW v2.0 online tool^2^. The *CHSA* nucleotide sequences resulted through cloning were deduced into amino acid sequences via EMBOSS Transeq^3^. Simultaneously, the amino acid sequences of *CHSA* gene from different insect species in lepidopteran order were collected from NCBI database and multiple sequence alignment was carried out in BioEdit software using ClustalW option.

To know the relatedness of *CHSA* gene among the lepidopteran insects, the phylogenetic analysis was conducted using MEGA v5.05 software (Tamura et al., 2013). A bootstrap analysis was done, and robustness of each cluster was verified in 1000 replicates. The nucleotide sequences of *CHSA* gene were used from different insects of lepidopteran order, viz., *B. mori* (Accession No: JQ320074), *Choristoneura fumiferana* (Clemens) (Accession No: EU561238), *Cnaphalocrocis medinalis* (Guenée) (Accession No: KP000843), *Earias vitella* (Fabricius) (Accession No: JX444555), *Ectropis obliqua* (Prout) (Accession No: EU482034), *Helicoverpa armigera* (Hubner) (Accession No: KP939100), *Helicoverpa zea* (Boddie) (Accession No: AF229127), *Hyblaea puera* (Cramer) (Accession No: JQ289043), *Leucinodes orbonalis* (Guenée) (Accession No: JX461234), *Mamestra brassicae* (Linnaeus) (Accession No: GQ281761), *Manduca sexta* (Linnaeus) (Accession No: AY062175), *Mythimna separata* (Walker) (Accession No: KT948989), Ostrinia *furnacalis* (Accession No: EU376026), *Phthorimaea operculella* (Zeller) (Accession No: KU720384), *P. xylostella* (Accession No: AB271784), and *S. exigua* (Accession No: KT932387).

### Double-Stranded RNA (dsRNA) Synthesis

The dsRNA was synthesized from the particular region of *CHSA* gene (Accession No: JN003621.1) which did not show off-target effects using dsCheck online software^4^ (Naito et al., 2005). To this region, primers were designed and T7 promoter sequence (TAATACGACTCACTATAGGGAGA) was incorporated at the 5’ends (Table 1). The purified PCR products were used for *in vitro* transcription with MEGAscript RNAi kit (Ambion Life Technologies, United States). The dsRNAs were annealed by incubating at 37°C for 3.5 h, followed by slow cooling to room temperature. The annealed dsRNAs were treated with DNase I and RNase at 37°C for 1 h, purified, and stored at −20°C.

### Feeding Bioassays Against Target Insects

#### Bioassay Study With *SlCHSA*, *CpCHSA*, *PxCHSA*, *MvCHSA dsRNA*

The dose effect was determined by diluting *SlCHSA* dsRNA with diethyl pyrocarbonate (DEPC) treated water to give a final concentration of 1, 3, 5, 7, and 9 μg/μl and further standardized dose of 3 μg/larvae was used for bioassay studies considering the production cost and efficacy. Similarly, the time-course expression analysis of the target gene was performed from day 1 onward until the end of the experiment. The bioassay was performed against *S. litura, C. partellus*, *P. xylostella*, *M. vitrata*, single second instar larvae were treated individually with 3 μg *SlCHSA*, *CpCHSA*, *PxCHSA*, *MvCHSA* dsRNA, respectively. The dsRNA was overlaid on castor leaf, baby corn, cauliflower leaf, and lablab pod, respectively, and were maintained in 80 mm sterile plastic cups with agar base for providing moisture till the end of the experiment. For control, the same number of larvae fed on respective feed overlaid with DEPC treated water (control) and Novaluron (Rimon—1.25 ml/l, benzoylphenyl urea, inhibits chitin formation) used as positive control in the experiment. There were 20 biological replicates in each group and the observations, viz., larval body weight, lethality rate, were observed. The data were analyzed statistically by one-way analysis of variance (ANOVA) at significance level (0.05) using the STAR software (International Rice research Institute [IRRI], 2013).

### Transgenic Tobacco Plants Expressing Hairpin *dsSlCHSA*

**FIGURE 1:**
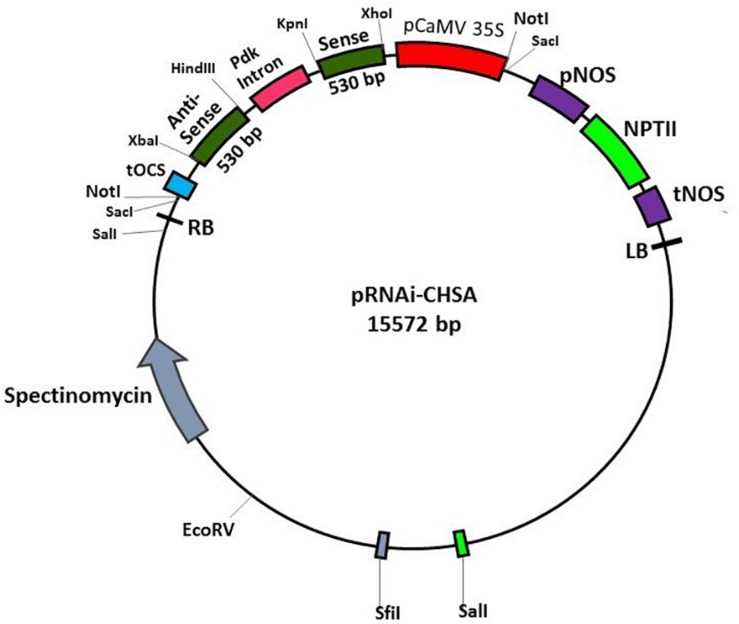
Physical map of pRNAi-CHSA vector.

The RNAi intermediate vector, pHANNIBAL (Source: CSIRO plant industry, Australia) contains RE site for directional insertion of PCR products on either side of the PDK intron. The 530 bp *SlCHSA* fragments cloned into pTZ57R/T vector were digested with *Hin*dIII and *Xba*I (*CHSA* antisense strand) and *Xho*I and *Kpn*I (*CHSA* sense strand), and subsequently inserted on either side of intron present in pHANNIBAL vector. The 3.0 kb RNAi-cassette was released from the cloned pHANNIBAL vector by *Not*I digestion, which was then cloned into pART27, plant transformation vector. The constructed pART27 expressing hairpin *SlCHSA* dsRNA (pRNAi-CHSA) contains a CaMV35S promoter, a sense strand of *SlCHSA*, a 741 bp intron, an antisense strand of *SlCHSA*, and an NOS terminator (Figure 1 and Supplementary Figure 1). *Agrobacterium tumefaciens* strain LBA4404 containing the binary plasmid pRNAi-CHSA was used for tobacco transformation. The plant transformation was done using standardized protocol and transformants were selected using 100 mg/l Kanamycin on MS medium (Horsch et al., 1985). After 1 month, rooted tobacco plants were transferred to greenhouse. Genomic DNA was isolated from the putative tobacco transformants and wild-type (WT) and checked by PCR using gene specific primers.

### Bioassay Study With Transgenic Tobacco Plants Expressing Hairpin *dsSlCHSA*

To assess the effects of transgenic tobacco plants expressing *dsSlCHSA*, on *S. litura* growth, second instar larvae (ten biological replicates) were reared on detached mature leaves (∼45 days old) of the control-WT, and five transgenic tobacco plants (dsSlCHSA E4, E5, E6, E9, and E11) and were weighed, respectively, for 7 days. Small leaf discs with 3 cm diameter were maintained in an 80 mm sterile plastic cup with agar base for providing moisture, and the observations were carried out.

### Assessment of Effect of dsRNA on the Target Gene Expression

#### RT-qPCR Analysis

Total RNA was extracted and pooled from three to five treated and untreated larvae in three groups independently and considered as three biological replicates. The reverse transcription product (100 ng) was used to perform the RT-qPCR amplification on CFX Connect^TM^ Real-Time PCR Systems (Bio-Rad, United States) using primers specific to *CHSA* gene (Table 1); 20 μl RT-qPCR reaction mixture included 1 μl of cDNA, 10 μl of 2X iQ^TM^ SYBR Green supermix (Bio-Rad, United States), 100 nM of primers, and nuclease free water to make up the total volume. The relative quantification was performed on CFX Connect^TM^ systems (Bio-Rad, United States) with the initial denaturation for 3 min at 95°C followed by 40 cycles at 95°C for 15 s and 55°C for 1 min. Each of the reactions was performed with three biological replicates, and the results were normalized with constitutively expressing *Actin* gene. RT-qPCR data were analyzed by using CFX Manager 2.1 (Bio-Rad, United States) software and was further verified using the standard delta-delta-Ct (ddCt) method.

## Results

### Sequencing and Phylogenetic Analysis of *CHSA* Gene

**FIGURE 2:**
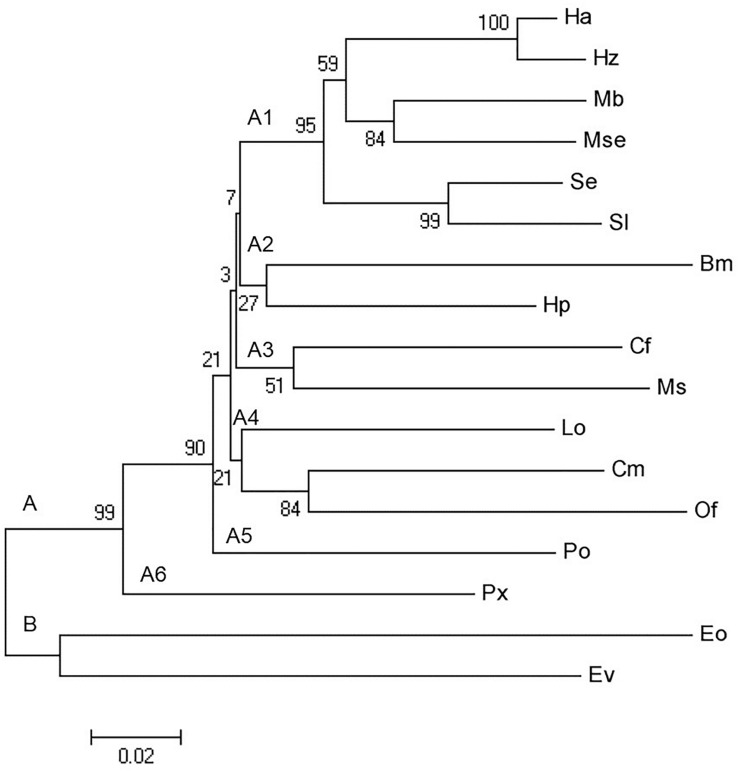
Phylogenetic tree for *chitin synthaseA* gene in different families of lepidopterans using MEGA v5.05 software. The scale on the *x*-axis represents the branch length and the numbers indicate the bootstrap values. *Chitin synthaseA* were from *Bombyx mori* (Bm), *Choristoneura fumiferana* (Cf), *Cnaphalocrocis medinalis* (Cm), *Earias vitella* (Ev), *Ectropis obliqua* (Eo), *Helicoverpa armigera* (Ha), *Helicoverpa zea* (Hz), *Hyblaea puera* (Hp), *Leucinodes orbonalis* (Lo), *Mamestra brassicae* (Mb), *Manduca sexta* (Ms), *Mythimna separata* (Mse), *O. furnacalis* (Of), *Phthorimaea operculella* (Po), *Plutella xylostella* (Px), *Spodoptera exigua* (Se), and *Spodoptera litura* (Sl). The accession numbers for various *chitin synthaseA* used in the phylogenetic analysis are provided in Section “Materials and Methods.”

The multiple sequence alignment of the cloned partial sequence of *CHSA* gene of *S. litura*, *C. partellus*, *P. xylostella*, and *M. vitrata* with the reference sequence of *S. litura CHSA* (Accession No: JN003621.1) showed match of 518 bp, as of expected size (Supplementary Figure 2). There were nucleotide variation at 143, 338, 355, 383, and 415 bp positions among the amplified sequence of *CHSA* from *S. litura*, *C. partellus*, *P. xylostella*, and *M. vitrata* and rest other sequences were similar to the reference sequence of *S. litura CHSA* (Supplementary Figure 3). The multiple sequence alignment of the deduced amino acid sequences of amplified *CHSA* from the four species with the other insects of lepidopteran order showed that most of the residues were conserved with 72.86% identity (Supplementary Figure 4). The phylogenetic tree formed two principle clusters, viz., A and B. Principle cluster A comprises of 13 genus of different families of Lepidoptera, viz., *B. mori*, *C. fumiferana*, *C. medinalis*, *Helicoverpa* sp., *H. puera*, *L. orbonalis*, *M. sexta*, *M. brassicae*, *M. separata*, *O. furnacalis*, *P. operculella*, *P. xylostella*, *Spodoptera* sp. Species from Noctuidae formed a separate subcluster A1 while *B. mori* (Bombycidae) and *H. puera* (Hyblacidae) formed subcluster A2 and showed the relatedness among them. *C. fumiferana* (Tortricidae) and *M. sexta* (Sphingidae) formed subcluster A3 while *L. orbonalis*, *C. medinalis*, *O. furnacalis* (Crambidae) formed subcluster A4. Notably, *P. operculella* (Gelechiidae) and *P. xylostella* (Plutellidae) falls in a separate subcluster A5 and A6 individually. Principle cluster B comprises two species *E. obliqua* (Geometridae) and *E. vitella* (Nolidae). All lepidopteran *CHSA* have a common lineage as high bootstrap value of 99 confirmed its phylogeny (Figure 2).

### Synthesis of dsRNA, Dose Effect, and Persistence of dsRNA on Target Gene

The dsRNA was synthesized from 518 bp of *SlCHSA* and “dsCheck” showed no off target gene candidate from selected region (Supplementary Figures 5, S6). NCBI-BLAST analysis of the nucleotide sequence of dsRNA also showed high level of sequence homology (99.00% identity) homology to *S. litura CHSA* gene and no significant homology to other genes of *S. litura* and other species.

The assessment of dose effect of dsRNA demonstrated that the concentration of 3–9 μg dsRNA is ideal for further bioassay (Supplementary Figure 7A). Bioassay results performed with standardized 3 μg/larvae dsRNA are presented and discussed. Time course expression analysis showed the maximum down-regulation at 48–72 h after treatment than 24 and 96 h after treatment (Supplementary Figure 7B).

### Bioassay Study With *SlCHSA* dsRNA

**FIGURE 3:**
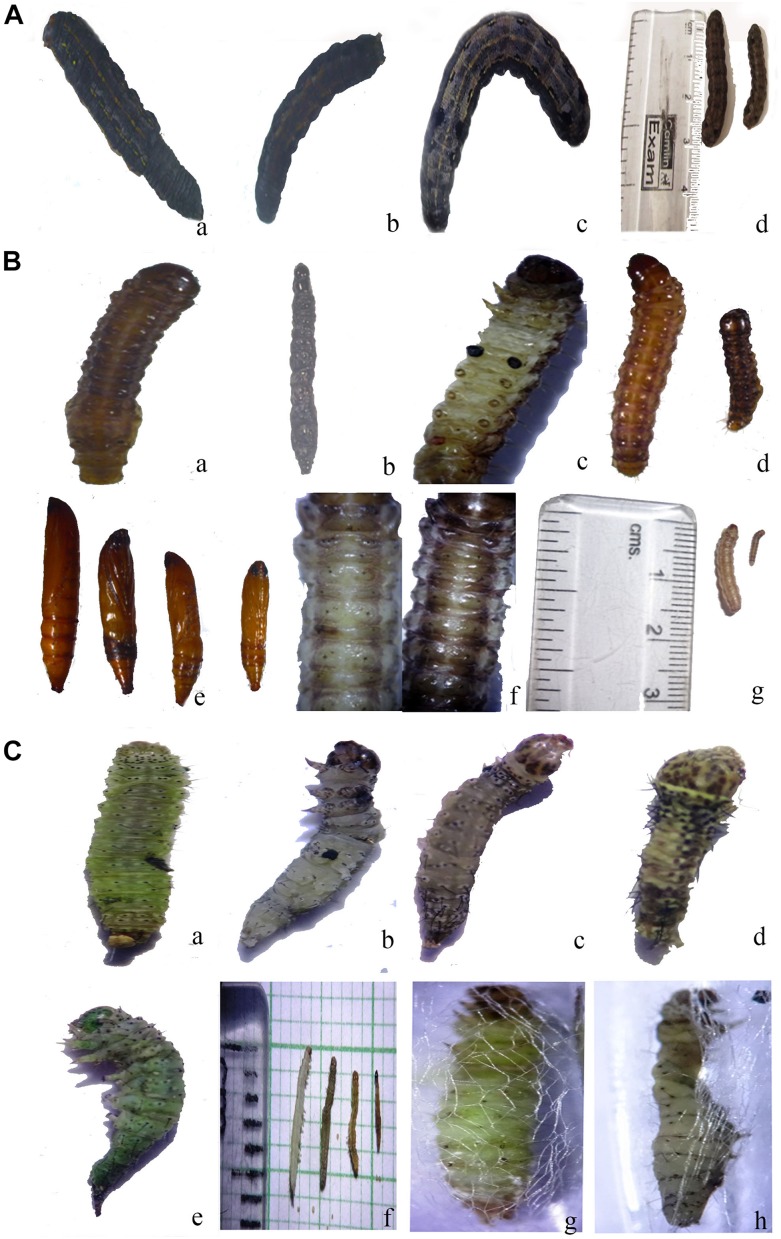
**(A)** Phenotype of *S. litura* after ingestion of *SlCHSA* dsRNA **(a)** “half ecdysis” phenotype of fourth instar larvae; **(b)** “black body” phenotype of fourth instar larvae; **(c)** normal phenotype of fourth instar larvae; **(d)** larvae of *S. litura*; control and treatment. **(B)** Phenotype of *C. partellus* after ingestion of *CpCHSA* dsRNA **(a)** “half ecdysis” phenotype of fourth instar larvae; **(b)** “black body” phenotype of fourth instar larvae; **(c)** pigmented spiracle; **(e)** abnormal phenotype at pupal stage; control and treatment; **(d,f,g)** larvae of *C. partellus*; control and treatment. **(C)** Phenotype of *P. xylostella* after ingestion of *PxCHSA* dsRNA **(a)** Control; **(b,c)** “black body” and melanized phenotype; **(d,e)** abnormal phenotype cramped, shrinked; **(f)** control and treated larvae; **(g)** control pupal stage and **(h)** abnormal pupal stage.

The *SlCHSA* dsRNA exhibited the lethal phenotypes like “Half- ecdysis” and “Black body.” The phenotype terminologies were used as per reported in *S. exigua* (Tian et al., 2009). Arrest in molting process also called as “Half- ecdysis” in which insects were not able to molt to next instar, was recorded in more than 80% of the larvae at 24–48 h after treatment. Notably, molting process was delayed by 24 h in dsRNA treated larvae as compared to control (DEPC treated H_2_O). About 25% larvae turned black at 48 h after treatment and designated as “Black body” phenotype or hyper pigmented phenotype (Figure 3A).

**FIGURE 4:**
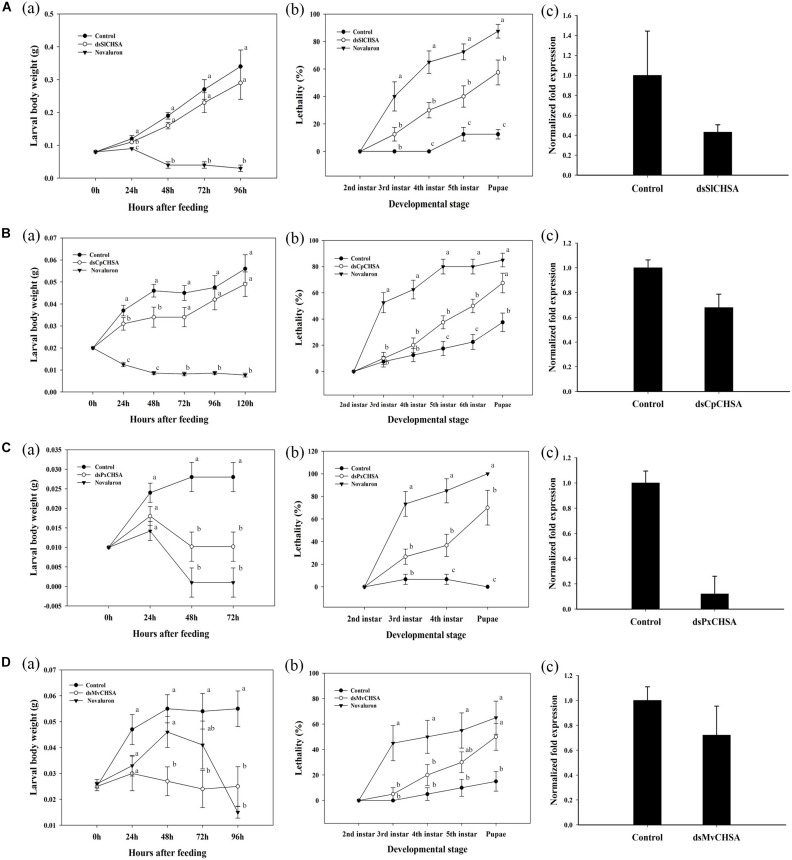
**(A)** Effect of ingested *dsSlCHSA* on *S. litura* larval body weight, lethality, and expression level. **(a)** The growth of *S. litura* after feeding *dsSlCHSA* was significantly delayed and larval weight reduced. **(b)** Ingestion of *dsSlCHSA* caused significantly higher lethality than in control. **(c)** *SlCHSA* mRNA level in *S. litura* was decreased after 96 h of feeding. **(B)** Effect of ingested *dsCpCHSA* on *C. partellus* larval body weight, lethality, and expression level. **(a)** The growth of *C. partellus* after feeding *dsCpCHSA* was significantly delayed and larval weight reduced. **(b)** Ingestion of *dsCpCHSA* caused significantly higher lethality than in control. **(c)** *CpCHSA* mRNA level in *C. partellus* was decreased after 120 h of feeding. **(C)** Effect of ingested *dsPxCHSA* on *P. xylostella* larval body weight, lethality, and expression level. **(a)** The growth of *P. xylostella* after feeding *dsPxCHSA* was significantly delayed and larval weight reduced. **(b)** Ingestion of *dsPxCHSA* caused significantly higher lethality than in control. **(c)** *PxCHSA* mRNA level in *P. xylostella* was decreased after 72 h of feeding. **(D)** Effects of ingested *dsMvCHSA* on *M. vitrata* larval body weight, lethality, and expression level. **(a)** The growth of *M. vitrata* after feeding *dsMvCHSA* was significantly delayed and larval weight reduced. **(b)** Ingestion of *dsMvCHSA* caused significantly higher lethality than in control. **(c)** *MvCHSA* mRNA level in *M. vitrata* were decreased after 96 h of feeding. Three biological replicates, each consist of pooled RNA from three to five larvae were used for analysis. The RT-qPCR data were analyzed using the delta–delta Ct method. Housekeeping gene, *Actin* used as internal control. The mRNA level in the treated group was relative to control group at the same time point. Error bars indicate standard error of mean. Statistical significance of difference was analyzed with ANOVA (*P* = 0.05). Means with different letters are significantly different (Tukey’s test).

The effect of *dsSlCHSA* on the growth and development of *S. litura* larvae was assessed by recording the larval body weight and lethality. The larval body weight reduction of 8.0% was observed at 24 h in *S. litura* fed with *dsSlCHSA* as compared to control. On the contrary, positive control (Novaluron) showed a significant reduction in larval body weight until pupation (Figure 4A). Similarly, lethality in *dsSlCHSA*-treated groups was calculated at different developmental stages. Lethality in dsRNA-treated groups continuously increased with time and lies from 12.5 to 57.5% while 40.0 to 87.5% in the positive control. Significant lethality was observed in fourth and fifth instar larvae of *S. litura* and its pupal stage. There were no lethal effects on growth and development of *S. litura* larvae fed with control (Figure 4A). The relative expression level using RT-qPCR analysis also showed the abundance of *SlCHSA* on treated larvae with 2.32-fold lesser expression than the control (Figure 4A).

### Bioassay Study With *CpCHSA* dsRNA

Similar, “half-ecdysis” and “black body” phenotypes were observed in *C. partellus* larvae at 48 h after treatment. In addition, the melanized spiracle, shrinked larvae, and abnormal pupae were also observed in the larvae fed with *CpCHSA* dsRNA and no lethal phenotypes in control (Figure 3B). The larval body weight was reduced by 26.00% at 24 and 48 h after treatment compared to control (Figure 4B). dsRNA-treated groups shown lethality from 10.0 to 67.5%, affecting significantly the later larval (fifth, sixth instar) and pupal stages while positive control showed obvious lethality of 52.0–85.0% (Figure 4B). Further, RT-qPCR analysis showed that the abundance of *CpCHSA* was 1.49-fold lower than the control (Figure 4B).

### Bioassay Study With *PxCHSA* dsRNA

The *PxCHSA* dsRNA resulted in “black body” phenotype in larval as well as in the pupal stage at 48–72 h after treatment. Similar, cramped, shrinked, melanized, hyperpigmented cuticle abnormalities were observed. Positive control also showed similar phenotypes (Figure 3C and Supplementary Figure 8). Notably, highest significant reduction of 64.00% in larval body weight was observed at 48 and 72 h after treatment (Figure 4C). Similarly, *dsPxCHSA*-treated groups showed increased lethality from 26.66 to 70.00 with significant lethal effects at fourth instar and pupal stage (Figure 4C). The results also correlate with the 8.33-fold decreased expression of *PxCHSA* in *dsPxCHSA*-treated group than the control (Figure 4C).

### Bioassay Study With *MvCHSA* dsRNA

In *M. vitrata*, about 50% of larvae turned black at 48 h after treatment and mortality was observed at 96 h after treatment while no lethal phenotypes were observed in control group. Significant differences in larval body weight was observed at 48, 72, and 96 h after treatment with 56% reduction in larval body weight compared to control (Figure 4D). Similarly, lethality in *dsMvCHSA*-treated groups was substantially increased from 5.0 to 50.0 and 52.5 to 85.0% in the positive control, at different developmental stages. Statistical analysis showed significant lethal effects at fifth instar and pupal stage of *M. vitrata* and the relative expression level of *MvCHSA* gene showed 1.38-fold lower in treated and control groups (Figure 4D).

### Molecular Analysis of Tobacco Transformants

A total of 45 independent putative transgenic tobacco shoots were recovered from 80 leaf explants after 4 weeks of co-cultivation with *A. tumefaciens* LBA4404 harboring pRNAi-CHSA construct. Fourteen of the 45 shoots were regenerated into whole plant and established in greenhouse. These established putative plants were used in further molecular analysis. Twelve of the 14 plants regenerated with pRNAi-CHSA construct found to be positive for the amplification of ∼296 and ∼530 bp partial sequences of *nptII* and *SlCHSA* genes. A similar band was observed in their respective positive control, pRNAi-CHSA, whereas untransformed control tobacco plants did not show any amplification (Supplementary Figure 9). The PCR positive tobacco plants were further validated using RT-qPCR which showed substantial amount of the *SlCHSA* expression level with highest in *dsSlCHSA* E4 plants (>4-fold change). The plants showing > 2-fold change (*dsSlCHSA* E4, E5, E6, E9, and E11) were selected for the bioassay studies (Supplementary Figure 10).

### Feeding Bioassay With Transgenic Tobacco Plants Expressing *dsSlCHSA*

**FIGURE 5:**
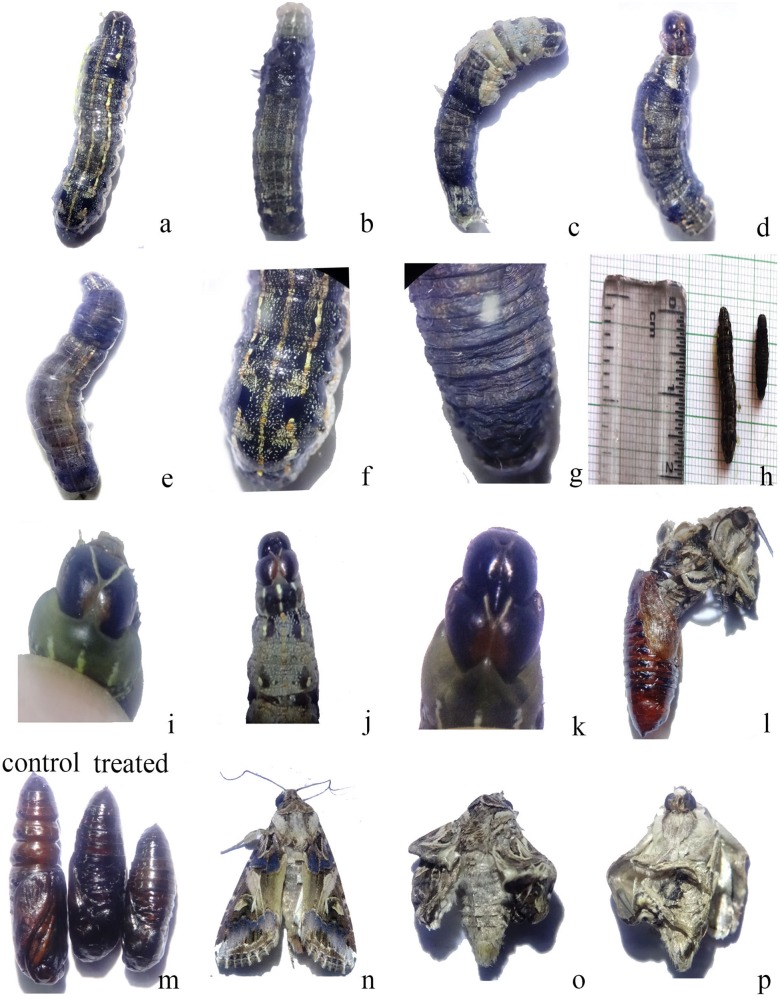
Phenotype of *S. litura* larvae after feeding with transgenic tobacco plants expressing *dsSlCHSA* **(a,f)** Control; **(b,e,g)** “black body” and melanized phenotype; **(c,d)** “half ecdysis” phenotype of fourth instar larvae; **(h)** larvae of *S. litura*; control and treatment; **(i)** Control larvae with normal head; **(j,k)** “double head” phenotype of fourth instar larvae; **(l)** lethal phenotype at pupal–adult intermediates; **(m)** abnormal phenotype at pupal stage; control and treatment; **(n)** normal phenotype at adult stage; **(o,p)** lethal phenotype (crimpled, weak cuticle with not spreading wings) at adult stage.

**FIGURE 6:**
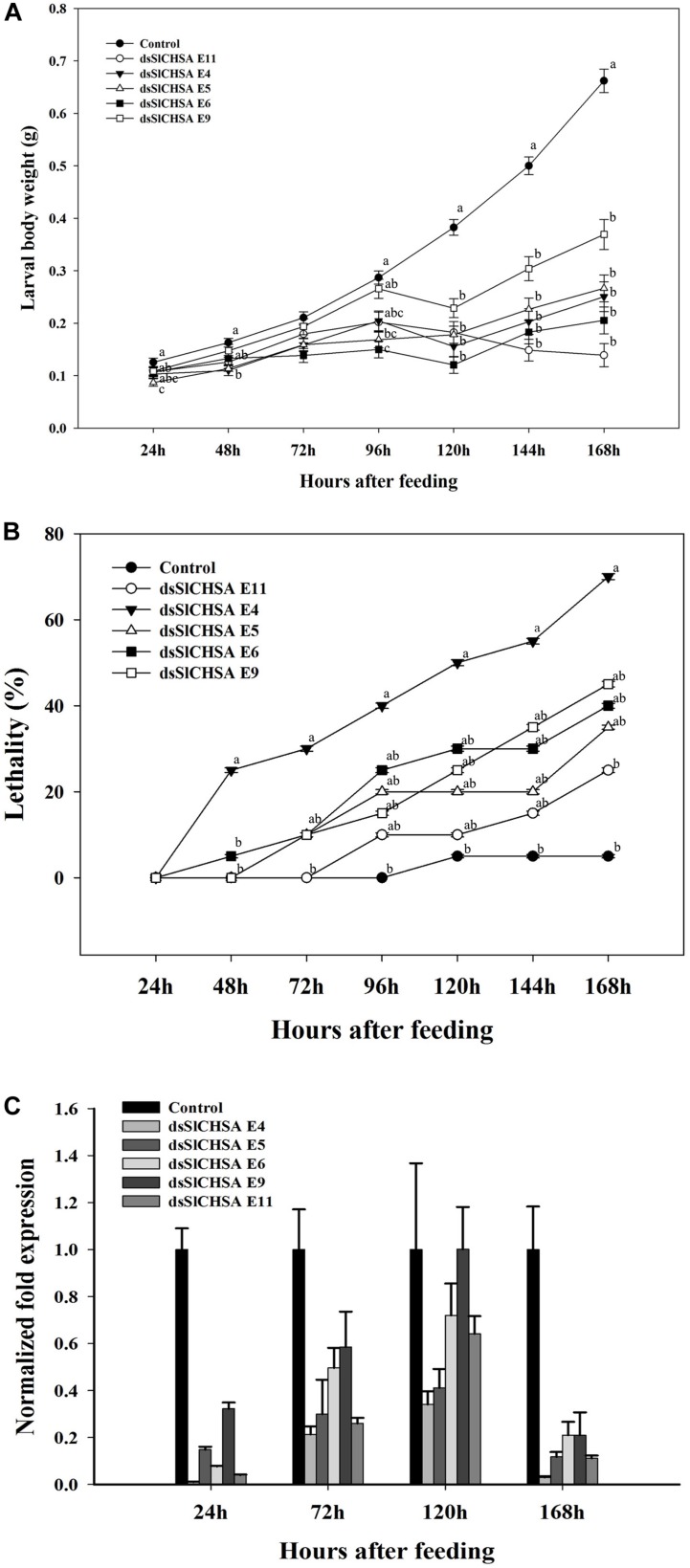
Effect of ingestion of transgenic tobacco plant tissues expressing *dsSlCHSA* on *S. litura* larval body weight, lethality, and expression level. **(A)** The growth of *S. litura* after feeding detached mature tobacco leaves expressing *dsSlCHSA* (E4, E5 E6, E9, and E11). **(B)** Feeding caused significantly higher lethality than in control. **(C)** *SlCHSA* mRNA level in *S. litura* at 24, 72, 120, and 168 h after feeding. Three biological replicates, each consist of pooled RNA from three to five larvae were used for analysis. The RT-qPCR data were analyzed using the delta–delta Ct method. Housekeeping gene, *Actin* used as internal control. The mRNA level in the treated group was relative to control group at the same time point. Error bars indicate standard error of mean. Statistical significance of difference was analyzed with ANOVA (*P* = 0.05). Means with different letters are significantly different (Tukey’s test).

Insect feeding trials with detached mature transgenic tobacco leaves expressing *dsSlCHSA* dsRNA showed lethal phenotypes such as “Half ecdysis,” “Black body,” and “Double head” at larval stage. Some abnormal phenotypes were also observed at pupal, pupal–adult intermediates, and adult stage (Figure 5). Significant reduction in larval body weight was observed at 120, 144, and 168 h after feeding of transgenic tobacco plants (*dsSlCHSA* E4, E5, E6, E9, and E11) as compared to WT (Figure 6A). Moreover, lethality was found more in larvae fed with *dsSlCHSA* E4 plants (25–70%) relative to WT (5.0%) (Figure 6B). RT-qPCR analysis had shown the varied amount of down-regulation of *SlCHSA* gene in insects at 24, 72, 120, and 168 h after feeding with an evident reduction in larvae fed with *dsSlCHSA* E4 (3–100-fold change) and *dsSlCHSA* E11 (1.5–33-fold change) transgenic tobacco plants (Figure 6C).

## Discussion

RNAi technologies hold great promise for the management of insect pests. Ingestible dsRNAs/siRNAs targeting key insect genes triggered through RNAi lead to growth inhibition, developmental aberrations, reduced fecundity, and mortality. However, previous reports have shown great variability and inconsistency with the targeted insect species, targeted gene, and mode of administration of RNAi in lepidopterans from time to time (Yang and Han, 2014). This study offers new insights to explore the potential of dsRNA-transgenic plant-mediated RNAi and design strategy for management of four key lepidopteran crop pests, viz., *S. litura*, *C. partellus*, *P. xylostella*, and *M. vitrata.*

In the present study, sequencing of partial *CHSA* gene from *S. litura*, *C. partellus*, *P. xylostella*, and *M. vitrata* has demonstrated that it forms an integral role in almost all crop pest, they are highly conserved among different species at genus level and with minor degree of variation at species level. Also, phylogenetic tree constructed using protein sequences of *CHSA* gene from different lepidopteran order is consistent with the inferred phylogeny of these larvae based on DNA sequences. Further, multiple sequence alignment and multi-protein alignment validated the sequence similarity of 72.86% for *CHSA* gene among the different lepidopteran species. Together signifying that, *CHSA* gene which is conserved in all insects of lepidopteran order is also one of the ideal targets for RNAi suppression.

### Dose Effect and Persistence of dsRNA

RNAi efficiency depends on the concentration of dsRNA (Chen et al., 2008). It differs in different order of insects (Tian et al., 2009). In earlier studies, the amount of dsRNA injected into lepidopteran insect ranged from 0.1 to 6 μg/larva (Rajagopal et al., 2002; Turner et al., 2006; Chen et al., 2008). As the earlier studies were based on injection method, different from the direct dsRNA feeding method, the dose effect of 1, 3, 5, 7, 9 μg/larvae dsRNA concentration on second instar *S. litura* was studied. RT-qPCR analysis exhibited the detectable amount of down-regulation at all the concentrations, but it was more significant at 3, 5, 7, 9 μg/larvae concentrations. Furthermore, the higher concentration did not make any difference; it was constant for 3–9 μg/larvae concentrations, revealing dose saturation effect. However, considering the cost of *in vitro* dsRNA synthesis and to avoid off-target effects with excessive dsRNA, we had employed 3 μg/larvae. These results were different from an earlier study on *H. armigera* where a linear increase in down-regulation was observed from 0.4 to 10 μg dsRNA concentrations and showed dose saturation effect at 53 μg/day (Yang and Han, 2014).

Simultaneously, we demonstrated the higher effect of dsRNA on the persistence of RNAi at time intervals of 48 and 72 h in comparison to 24 and 96 h after treatment. This suggested that single application of dsRNA triggers RNAi at 48 h and sustained till 72 h after feeding. Asokan et al. (2013) also observed single application of dsRNA resulted in delayed and transient silencing, while multiple applications led to an early onset and sustained silencing in case of chymotrypsin and jhamt.

### dsRNA-Transgenic Plant-Mediated RNAi Resulted in Lethal Phenotypes

The knockdown of *SlCHSA*, *CpCHSA*, *PxCHSA*, and *MvCHSA* through dsRNA in *S. litura, C. partellus*, *P. xylostella*, and *M. vitrata* respectively, documented lethal phenotypes like “Half ecdysis” and “Black body” at larval stage, which is consistent with the earlier study done in *S. exigua* (Tian et al., 2009; Li et al., 2013). On the contrary, we have also demonstrated significant change in pupal phenotype in *C. partellus* and *P. xylostella* though they did not complete the pupation stage and were not able to emerge out as adults. Similar observation was reported by Arakane et al. (2005) in *T. castaneum* larvae injected with dsRNA in the penultimate larvae which failed to pupate and did not complete pupal development. However, the insect pests targeted in our study are destructive at larval stage and control of them at early larval stage is important. The positive control Novaluron belonging to benzoylphenyl urea group and acts as an insect growth regulator by inhibiting the biosynthesis of chitin, also showed similar phenotypes suggesting dsRNA might have alike mode of action to that Novaluron. Retnakaran and Wright (1987) also observed similar phenotypes in insect treated with acrylureas which also belongs to benzoylphenyl urea group and acts as a selective disrupter of chitin synthesis in insects. Apparently, no lethal phenotypes were observed in the control group, so it was unlikely that these phenotypes were caused by injury or infection. Besides that, molting was delayed ∼24 h in the RNAi-treated group as compared to control larvae which molted normally into next larval instar.

Nevertheless, *in vitro* synthesized dsRNA is not applicable for the management of insect pest in the fields because of high concentration of dsRNA is required to cause severe RNAi effects as they are degraded in the digestive system (Zhu et al., 2012). Therefore, it is important to develop an efficient method of delivery for large scale pest control in the fields. Transgenic technology has generated insect resistant plants to reduce yield loss and utilization of pesticides (Kos et al., 2009). However, there is continuous development of insect resistance and outbreak of non-target pests (Lu et al., 2010; Jin et al., 2015). Thus, transgenic plants expressing suitable insect dsRNA can be exploited to control insect pests. To determine its potential over the direct dsRNA feeding, we constructed pRNAi-CHSA vector to express *dsSlCHSA* genes and validated in tobacco for mRNA abundance of the transgene, and *S. litura* larvae were evaluated for down-regulation of *CHSA* gene expression. Tobacco and *S. litura* were used as plant and insect model system, respectively, for the preliminary studies and the generation of transgenic plants specific for other insects is future line of work. Our results suggested that *S. litura* larvae fed with leaves of transgenic tobacco plants expressing *dsSlCHSA* manifested same lethal phenotypes such as “Half ecdysis,” “Black body,” and “Double head phenotype” leading to mortality. These phenotypes were like those reported in *S. exigua* after feeding bacterially expressed dsRNA of *SeCHSA* gene (Tian et al., 2009) but they did not observe any abnormality at later developmental stages which was different from our observations. We also observed abnormal phenotypes (crimpled) in pupal, pupal–adult intermediates, and adult stage as reported in *H. armigera* (Zhu et al., 2012) and partly in agreement with insects of other orders, viz., *T. castaneum* (Arakane et al., 2005, 2008), *L. migratoria* (Zhang et al., 2010; Liu et al., 2012) and *N. lugens* (Wang et al., 2012). Importantly, the lethal phenotypes observed in larva–pupa stage and pupa–adult stage were in agreement with the earlier report in *S. exigua* targeting trehalase gene (*SeTre-1*) which inhibits the expression of *CHSA* gene (Chen et al., 2010). These lethal phenotypes were more pronounced compared to phenotypes observed with direct application of dsRNA.

### dsRNA-Transgenic Plant-Mediated RNAi Affecting Growth and Development

Reduction in larval body weight at different times period among the four lepidopteran species suggested the time sensitivity of dsRNA molecules, which may reach at threshold level (required to inhibit the target) at different time depending on the species. The *S. litura* larvae showed less reduction in larval body weight compared to *C. partellus*, *M. vitrata*, and *P. xylostella*. One possible reason could be the variation in the physiological conditions of the gut fluids or the presence of dsRNAase in the digestive tract of insects. Still the mechanism underlying the variability is not well understood in lepidopterans and it is likely that a lot remains to be discovered. Moreover, the growth pattern of the RNAi-treated group was more like Novaluron treated group compared to control in *P. xylostella* and *M. vitrata*, with a drastic reduction at the later developmental stages. This presents an opportunity for molecular biologists in developing insect-specific molecular biopesticides using dsRNA. To our knowledge, this is only study where commercially available product for controlling insect pests has been used for the comparative studies. Another factor measured lethality demonstrated higher effects during late developmental stages in all four lepidopteran spp. A previous study in lepidopteran *M. separate* also reported effects at later developmental stages with only 10–16% mortality using bacterially expressing chitinase gene (Ganbaatar et al., 2017). We showed higher lethality in *dsPxCHSA* treated group and *S. litura* fed with leaves of transgenic tobacco plants expressing *dsSlCHSA* (dsSlCHSA E4) (∼70%). The down-regulation of *CHSA* gene did not yield cent percent lethality or phenotypic variation in all the four insects, which may be due to the incomplete down-regulation of *CHSA* gene and some amount of *CHSA* transcript might have translated to produce chitin.

### dsRNA-Transgenic Plant-Mediated RNAi Reduces *CHSA* mRNA Level

Expression analysis through RT-qPCR showed decreased *CHSA* mRNA level in all the four lepidopterans. Higher down-regulation of *CHSA* was observed in *dsPxCHSA* and transgenic plant-mediated RNAi, affecting all molting stages (larval–larval, larval–pupal, and pupal–adult) and cuticular chitin synthesis. A previous study in *S. litura* using injection of dsRNA did not cause lethal phenotypes and lethality, in spite of reduction in expression level (Rajagopal et al., 2002). We also demonstrated that transgenic tobacco plants expressing *dsSlCHSA* showed positive correlation between the mRNA abundance of *SlCHSA* and percent lethality, which was further validated by RT-qPCR analysis that showed higher down-regulation of *SlCHSA* gene in insects fed on leaves of dsSlCHSA E4 plant. Such positive correlation between expression level of Cry protein and insect lethality have been also reported in many studies (Bhattacharya et al., 2002; Estrada et al., 2007; Ramu et al., 2012). Furthermore, the bell-shaped trend of normal *CHSA* gene expression remains the same after down-regulation of *CHSA*, which increases continually from first to the last instar and decreases from pupal to the adult stage. This exhibited stage specific and target specific manner of RNAi efficacy. Therefore, stage of insect to be targeted based on target gene expression and selection of target genes are the critical considerations before commencing RNAi experiments.

## Conclusion

RNAi is a sequence-specific gene silencing mechanism mediated by dsRNA, which has been harnessed as a useful tool in devising novel insect pest management strategies. Our study demonstrates efficacy of dsRNA-transgenic plant-mediated RNAi in reducing mRNA transcript level of specific targeted gene causing lethal phenotypes, reduction in larval body weight, and eventually mortality. Although the inconsistencies lie between the insects belonging to same order, targeting same gene nevertheless the technology has proved its potentiality. *CHSA* targeted in present study is as an ideal target gene and presents an effective alternative opportunity for designing broad spectrum biopesticide for the management of insect pests.

## Data Availability Statement

All datasets generated for this study are included in the article/Supplementary Material.

## Author Contributions

SR conducted the experiments and wrote the manuscript. SR and AR analyzed the data. SM and KK conceptualized and supervised the project. AR, SM, and KK edited the manuscript. All authors read and approved the final document.

## Conflict of Interest

The authors declare that the research was conducted in the absence of any commercial or financial relationships that could be construed as a potential conflict of interest.

## Footnotes

1 https://www.ncbi.nlm.nih.gov/

2 www.ebi.ac.uk/Tools/msa/clustalw2/

3 http://www.ebi.ac.uk/Tools/st/emboss_transeq

4 http://dsCheck.RNAi.jp/
