# Supplementary material for: Comparative Analysis of Chitin SynthaseA dsRNA Mediated RNA Interference for Management of Crop Pests of Different Families of Lepidoptera
Source: Front Plant Sci. 2020 Apr 17;11:427. doi: 10.3389/fpls.2020.00427 (PMC7182115; doi:10.3389/fpls.2020.00427)
Supplement: Supplementary file 1 [file Data_Sheet_1.doc]

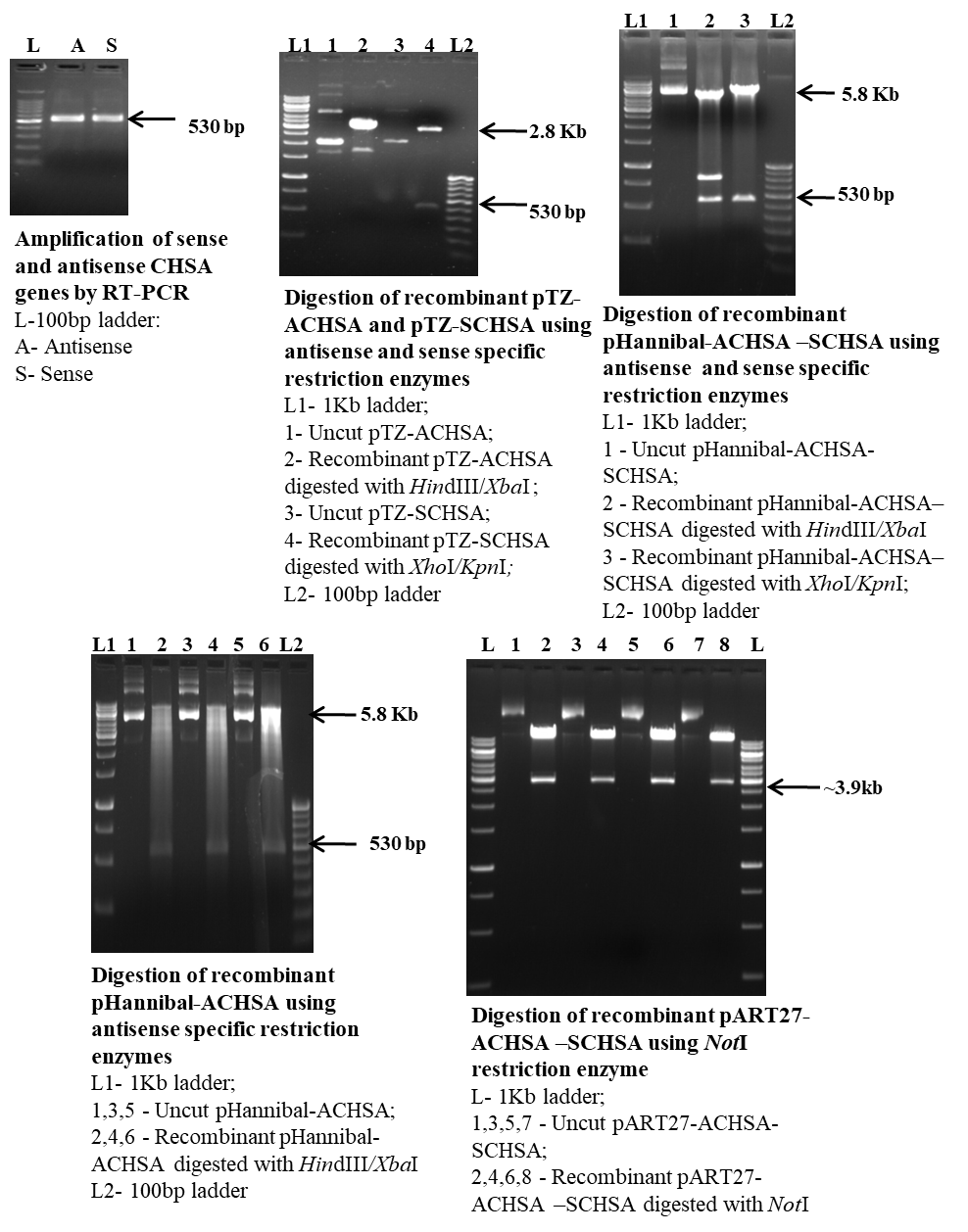


**Supplementary Figure 1.** Construction of RNAi vector with *SlCHSA* gene


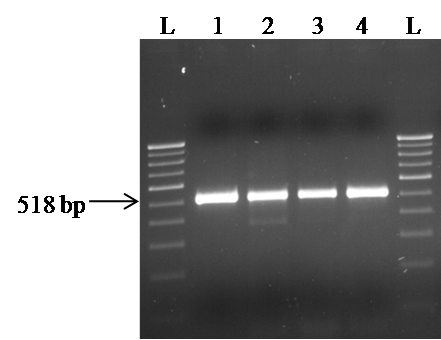


**Supplementary Figure 2.** PCR amplification of partial *SlCHSA* gene from cDNA

Lane L-100 bp DNA ladder, Lane 1- 4: *SlCHSA* gene amplification in *S. litura*, *C. partellus*, *P. xylostella*, *M. vitrata.*


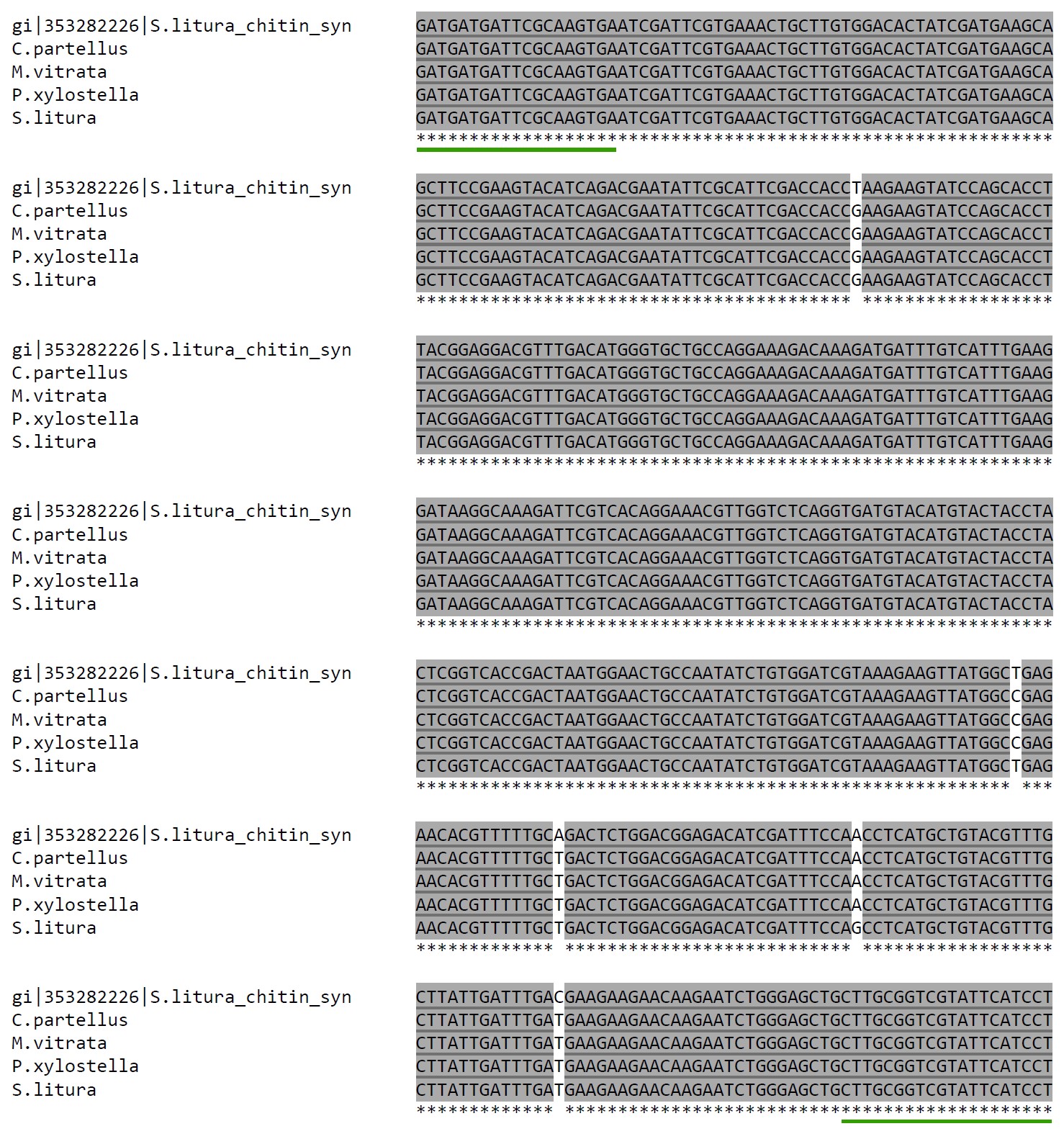


**Supplementary Figure 3.** Multiple sequence alignment of the partial sequence of *CHSA* gene in *S. litura, C. partellus, P. xylostella* and *M. vitrata* with reference sequence of *S. litura* *CHSA* (Acc. no. JN003621.1) using ClustalW software. Grey shades represent completely conserved bases. Primer sequences used for amplification of partial *CHSA* gene was underlined by the green line.


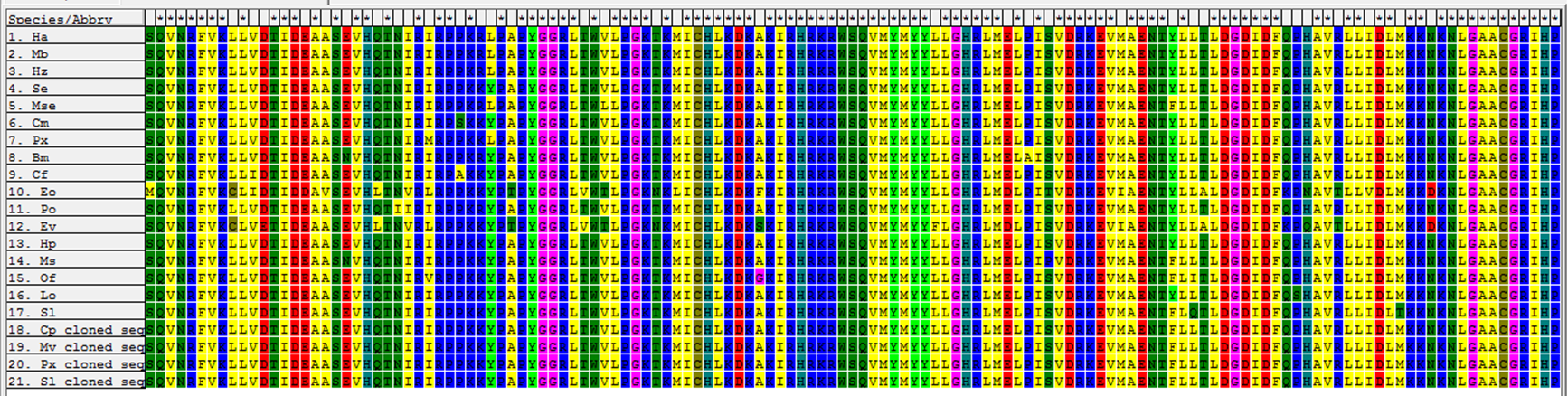


**Supplementary Figure 4.** Multiple sequence alignment of the deduced amino acid sequence of partial *chitin synthaseA* gene in different families of lepidopterans using MEGA v5.05 software. The Sl, Cp, Px, Mv cloned seq (*S. litura*, *C. partellus, P. xylostella* and *M. vitrata* respectively) indicates the sequences (template sequence) generated in this study. The other *Chitin synthaseA* were from *Bombyx mori* (Bm), *Choristoneura fumiferana* (Cf), *Cnaphalocrocis medinalis* (Cm), *Earias vitella* (Ev), *Ectropis obliqua* (Eo), *Helicoverpa armigera* (Ha), *Helicoverpa zea* (Hz), *Hyblaea puera* (Hp), *Leucinodes orbonalis* (Lo), *Mamestra brassicae* (Mb), *Manduca sexta* (Ms), *Mythimna separata* (Mse), *O. furnacalis* (Of), *Phthorimaea operculella* (Po), *Plutella xylostella* (Px), *Spodoptera exigua* (Se) and *Spodoptera litura* (Sl). The accession numbers for various *chitin synthaseA* used in the sequence alignment are provided in the materials and methods section


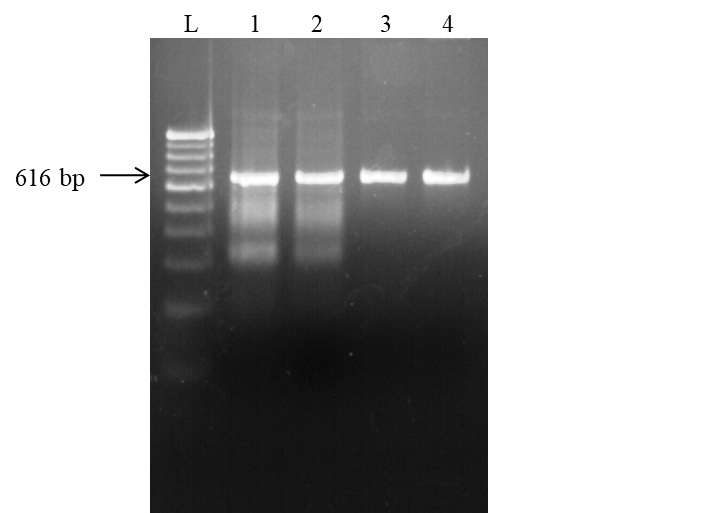


**Supplementary Figure 5. Synthesis of dsRNA**

Lane L-100 bp DNA ladder, Lane 1- 4: dsRNA for *CHSA* from *S. litura*,
*C. partellus*, *P. xylostella* and *M. vitrata*


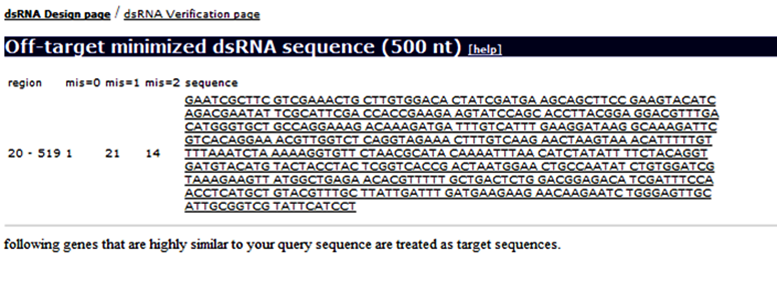


**Supplementary Figure 6.** Off-target minimized *CHSA* dsRNA sequences- *dsCheck*


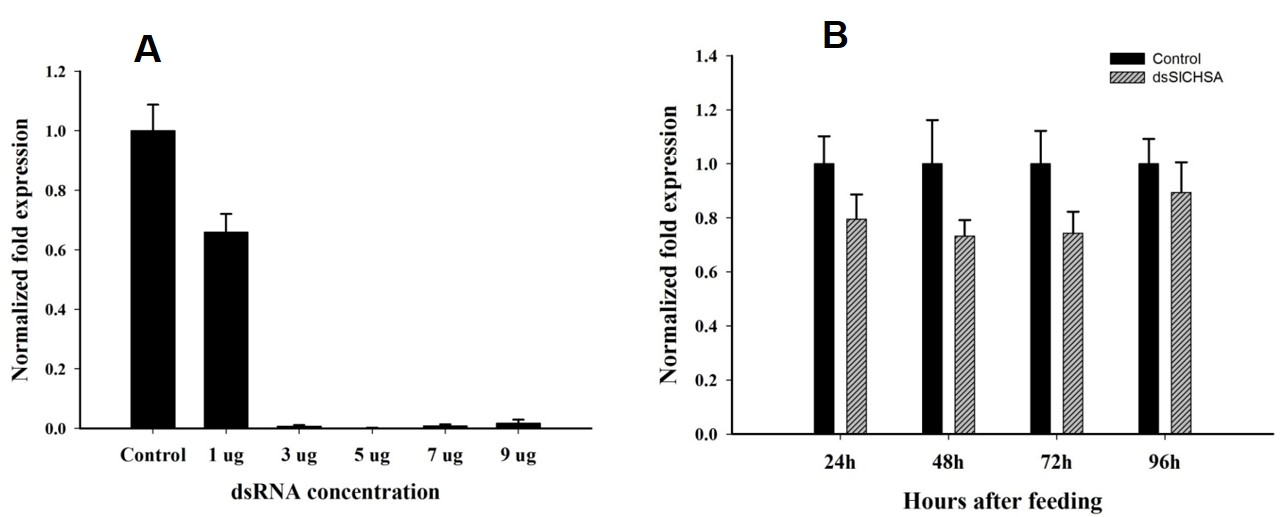


**Supplementary Figure 7 (A)** Dose effect of double-stranded RNA (dsRNA) on mRNA level of *SlCHSA* gene in the *S. litura* larvae. The insects were reared on diet containing a different concentration of dsRNA for 120 h. (**B)** Effect of double-stranded RNA (dsRNA) on mRNA level of *SlCHSA* gene in the *S. litura* larvae at different time intervals. The insects were reared on diet containing a 1μg concentration of dsRNA for 96 h. Three biological replicates, each consist of pooled RNA from three to five larvae were used for analysis. The RT-qPCR data were analyzed using the delta-delta Ct method. Housekeeping gene, *Actin* used as internal control. The mRNA level in the treated group was relative to control group at the same time point. Error bars indicate standard error of mean


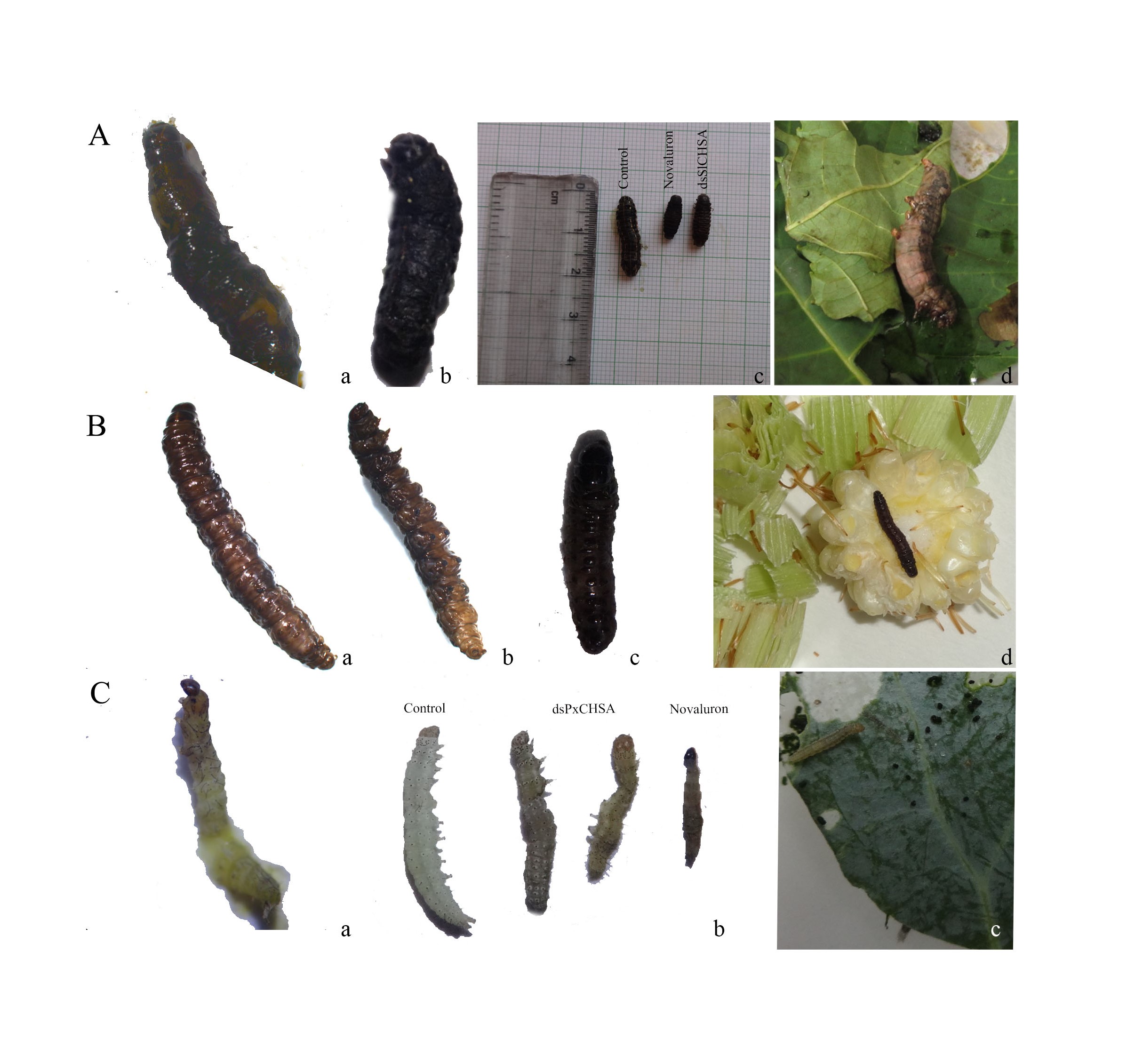


**Supplementary Figure 8 (A)** Phenotype of *S. litura* after ingestion of Novaluron (a, b) “Half ecdysis” and “Black body” phenotype of 4^th^ instar larvae; (c) Larvae of *S. litura;* control and treatment; (d) Abnormal phenotype of 4^th^ instar larvae. **(B)** Phenotype of *C. partellus* after ingestion of Novaluron (a, b, c, d) “Black body” phenotype of 4^th^ instar larvae; (b) Pigmented spiracle. **(C)** Phenotype of *P. xylostella* after ingestion of Novaluron (a, c) “Black body” phenotype; (b) Larvae of *P. xylostella;* control and treatment.


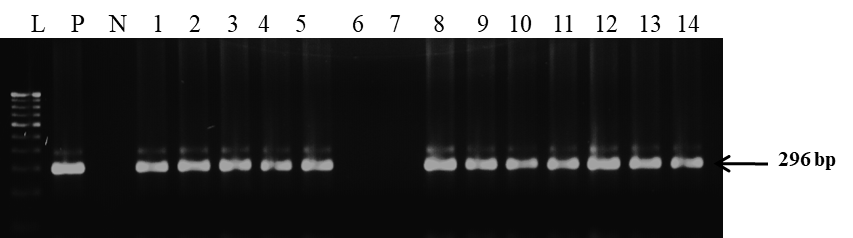

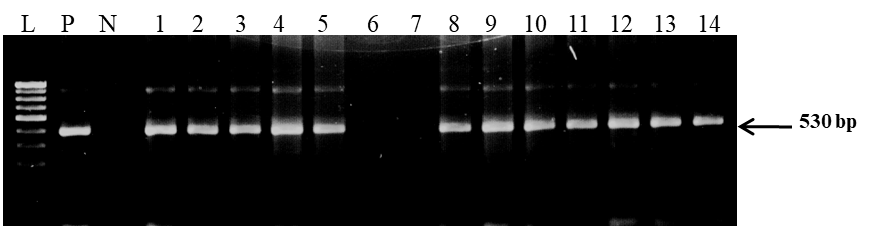


**A**

**B**


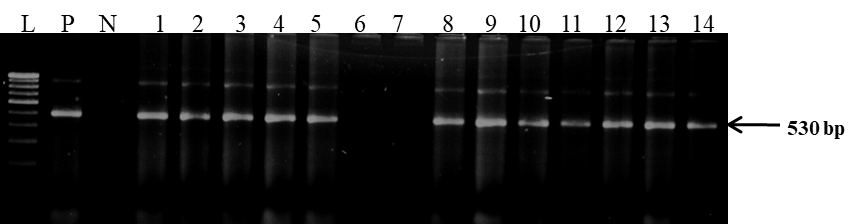


**C**

**Supplementary Figure 9.** PCR analysis of tobacco plants transformed with pART27 *CHSA* construct for the presence of (A) *nptII* gene sequence (B) antisense strand of *SlCHSA* gene (C) sense strand of *SlCHSA* gene

Lane L: 100 bp DNA ladder, Lane P: positive control (pART27 *CHSA*), Lane N: negative control (non transgenic plant), Lane 1- 14: putative transgenic plants


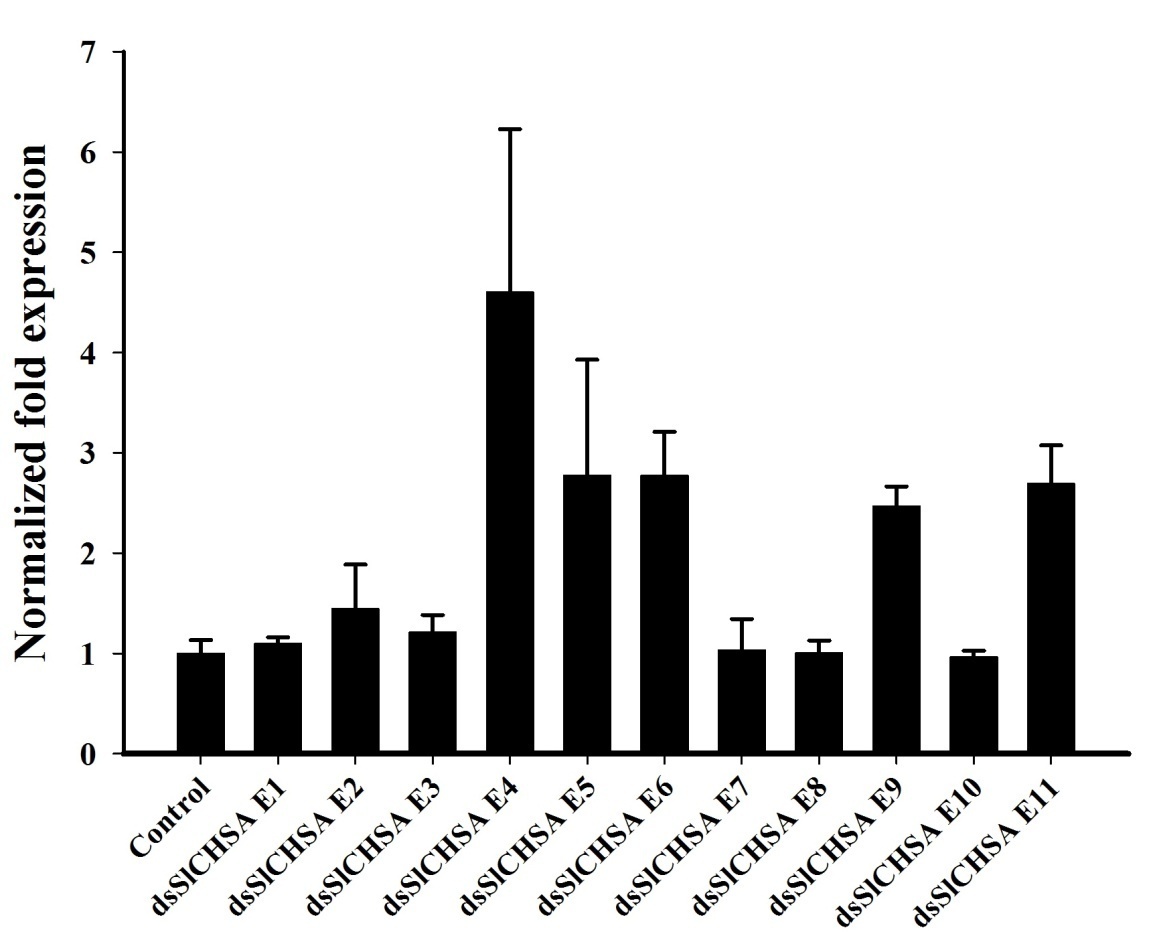


**Supplementary Figure 10.** *SlCHSA* expression in leaves of transgenic tobacco plants (E1 to E11)
